# Six different football shoes, one playing surface and the weather; Assessing variation in shoe-surface traction over one season of elite football

**DOI:** 10.1371/journal.pone.0216364

**Published:** 2019-04-30

**Authors:** Athol Thomson, Rodney Whiteley, Mathew Wilson, Chris Bleakley

**Affiliations:** 1 Aspetar Orthopaedic and Sports Medicine Hospital, Doha, Qatar; 2 University of Ulster, Jordanstown, County Antrim, United Kingdom; 3 High Point University, High Point, NC, United States of America; Mayo Clinic Rochester, UNITED STATES

## Abstract

**Introduction:**

An optimal range of shoe-surface traction (grip) exists to improve performance and minimise injury risk. Little information exists regarding the magnitude of traction forces at shoe-surface interface across a full season of elite football (soccer) using common football shoes.

**Objective:**

To assess variation in shoe-surface traction of six different football shoe models throughout a full playing season in Qatar encompassing climatic and grass species variations.

**Methods:**

Football shoes were loaded onto a portable shoe-surface traction testing machine at five individual testing time points to collect traction data (rotational and translational) on a soccer playing surface across one season. Surface mechanical properties (surface hardness, soil moisture) and climate data (temperature and humidity) were collected at each testing time point.

**Results:**

Peak rotational traction was significantly different across shoe models (F = 218, df = 5, p <0.0001), shoe outsole groups (F = 316.2, df = 2, p < .0001), and grass species (F = 202.8, df = 4, p < 0.0001). No main effect for shoe model was found for translational traction (F = 2.392, p = 0.07).

**Conclusions:**

The rotational (but not translational) traction varied substantially across different shoe types, outsole groups, and grass species. Highest rotational traction values were seen with soft ground outsole (screw-in metal studs) shoes tested on warm season grass. This objective data allows more informed footwear choices for football played in warm/hot climates on sand-based elite football playing surfaces. Further research is required to confirm if these findings extend across other football shoe brands.

## Introduction

Association football (soccer) is an invasion game involving multiple bouts of intermittent sprinting and directional changes. Elite footballers undertake 1500–3100 metres of high intensity running per match [1,2], with accelerations contributing 7–10% of the total player load, and decelerations contributing 5–7% [3]. A recent systematic review examining activity demands of team sports found that the highest volume of cutting movements occur in football, with players performing up to 800 cuts per game [4].

A player’s ability to accelerate, decelerate, and change direction is largely influenced by the available traction between the football shoe and playing surface [5,6]. Two important components of traction exist: *translational traction* which is the horizontal force required to overcome the resistance between the shoe outsole (studs) and playing surface; and *rotational traction* which is the rotational force required to release the studs through the playing surface in a rotational manner. Although increases in translational traction (straight line or side-to-side) are linked to improved performance (e.g., time to complete an agility course or acceleration task) [5,6], higher levels of rotational traction are linked to greater risk of lower limb injury [7–11].

Optimal shoe-surface conditions should therefore attenuate rotational resistance whilst maintaining translational traction or playing performance (no slipping for players) [7,10]. This is sometimes difficult to achieve as traction varies according to shoe outsole, stud/cleat configuration [12], and the characteristics of the playing surface [13,14], among other factors [5]. Further challenges arise based on the wide array of outsole designs currently on the market and intermittent changes in playing surface throughout a playing season [11,15].

Mechanical properties of natural grass playing surfaces are moderated by climatic factors such as. temperature and soil moisture. Surface hardness and subsequent penetration of the studs on the surface ultimately alters traction [11,16–20]. Varied shoe-surface interface conditions change a players muscle recruitment patterns [21], movement strategies [22], and injury risk [11,23].

Importantly, varied climatic conditions means certain geographical regions support certain species of grass. Moreover, different grass species have different mechanical properties [11,15]. For example, drought resistant warm season grass species are associated with increased risk of anterior cruciate ligament injury compared to other cool season grass species in Australian rules football [24]. This is attributed to higher shoe-surface traction with warm season grass species.

Portable testing devices can now be used to objectively measure mechanical properties of playing surfaces and quantify their interaction with shoe outsoles. These data could help to streamline decision making concerning the suitability of football shoe outsoles, allowing players to tailor their selection for given climatic or surface conditions. Our primary aim is to assess variation in shoe surface traction of different football shoes on one football playing surface throughout a season in Qatar. As a secondary objective, any moderating effects of temperature, humidity, soil moisture, and surface hardness are examined.

## Methods

### Playing surface

One natural grass football pitch (Qatar national team outdoor training pitch) with a sand rootzone and no hybrid reinforcement was tested at five time points over a single football season in Doha, Qatar (November 2017, January, March, April and May 2018).

The climatic conditions in Qatar mean that grass type consistently changes during a playing season: ranging from natural warm-season C_4_ grass (*Paspalum vaginatum* ‘Paspalum’) in summer months; to warm-season grass over-seeded with cool-season C_3_ grass (*Lolium perenne* ‘Perennial Rye’) in transition cooler months; to predominantly cool season (Perennial Rye) grass in the coldest month (January 2018). Warm season grasses are more heat and drought tolerant but become dormant at lower temperatures, thus the need to over-sow with cool-season grass [15].

Ground staff maintained 100% grass coverage with grass length at 25mm on the day of each test. Surface hardness was assessed using a 2.25kg Clegg hammer dropped from 450mm (SD instrumentation, England). Soil moisture (Delta-t ML2X/ML3 thetaprobe, England) and temperature/humidity (Kestrel 4400 heat stress tracker, USA) were also recorded. These surface tests were carried out in five different pitch locations and repeated five times in each location (moving to untested/unaffected grass for each test).

### Football shoe models

**Fig 1:**
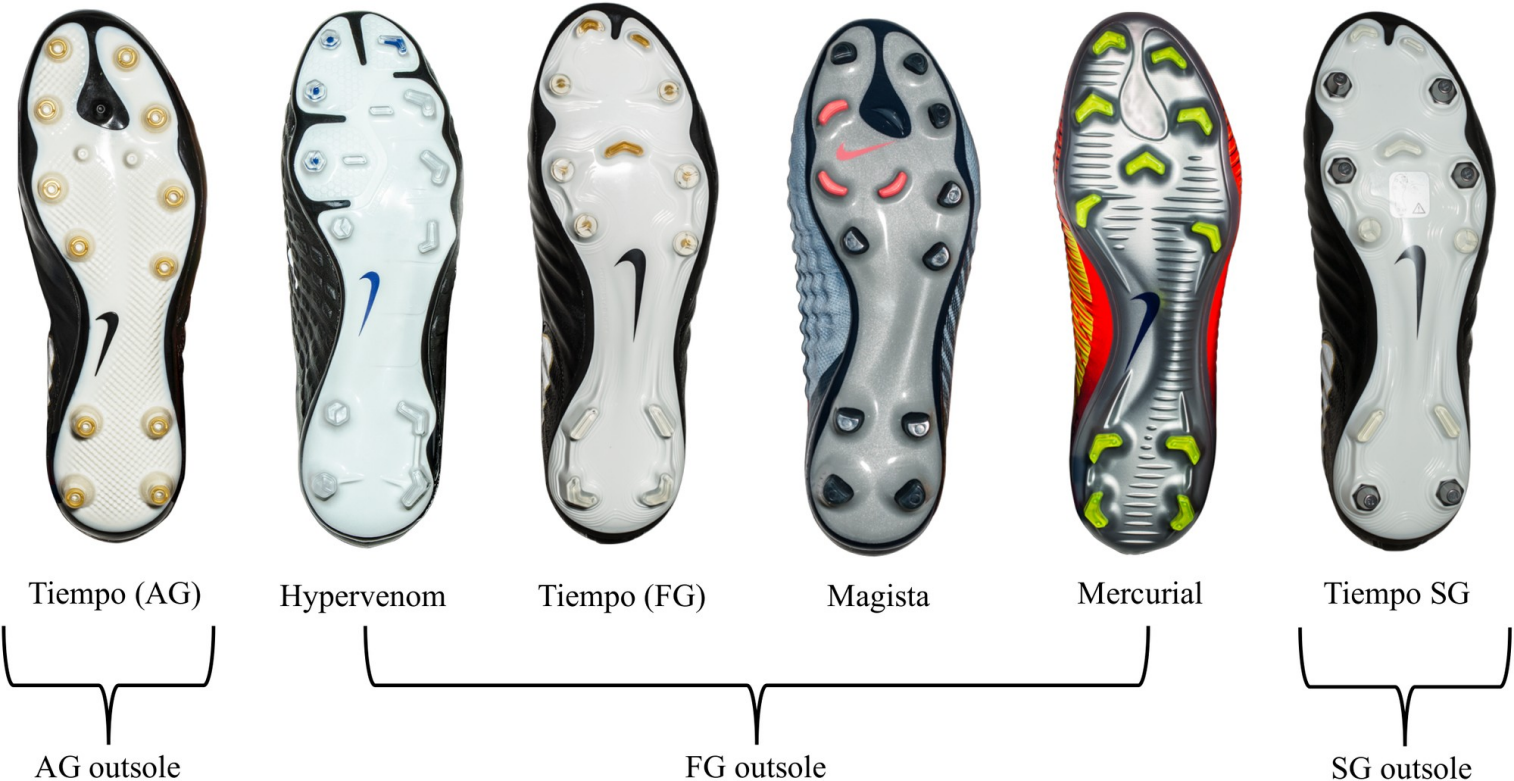
Football shoe models tested and outsole grouping. AG = artificial grass. FG = firm ground. SG = soft ground.

Six different football shoes manufactured by Nike (Beverton, Oregon USA) were tested. These consisted of one artificial grass (AG) outsole (Nike Tiempo legend VII Pro AG), four firm ground (FG) outsoles (Mercurial vapor XI FG, Magista obra II Elite FG, Tiempo legend VII FG, and Hypervenom Phantom III FG), and one soft ground (SG) outsole (Tiempo legend VII Pro SG) (Fig 1). According to a worldwide professional football shoe database (Footballbootsdb.com), our sample shoes included four of the six most used football shoes in the world.

### Outsole types

Shoes were grouped according to their outsole type for further analysis. Shoes are marketed and sold in these “silos” with players expected to select an outsole type that best suits the surface and climate conditions they will play on. Soft ground (SG) shoes have fewer, longer, conical (or tapered) metal “screw-in” studs for wet, muddy, or low surface traction conditions. Firm ground (FG) shoes have moulded cleats, blades, or round studs (not screw-in) that are generally used on firm, dry surfaces. Artificial grass (AG) shoes have several small, short, round moulded studs that are generally used on artificial turf.

### Shoe-surface traction testing

**Fig 2:**
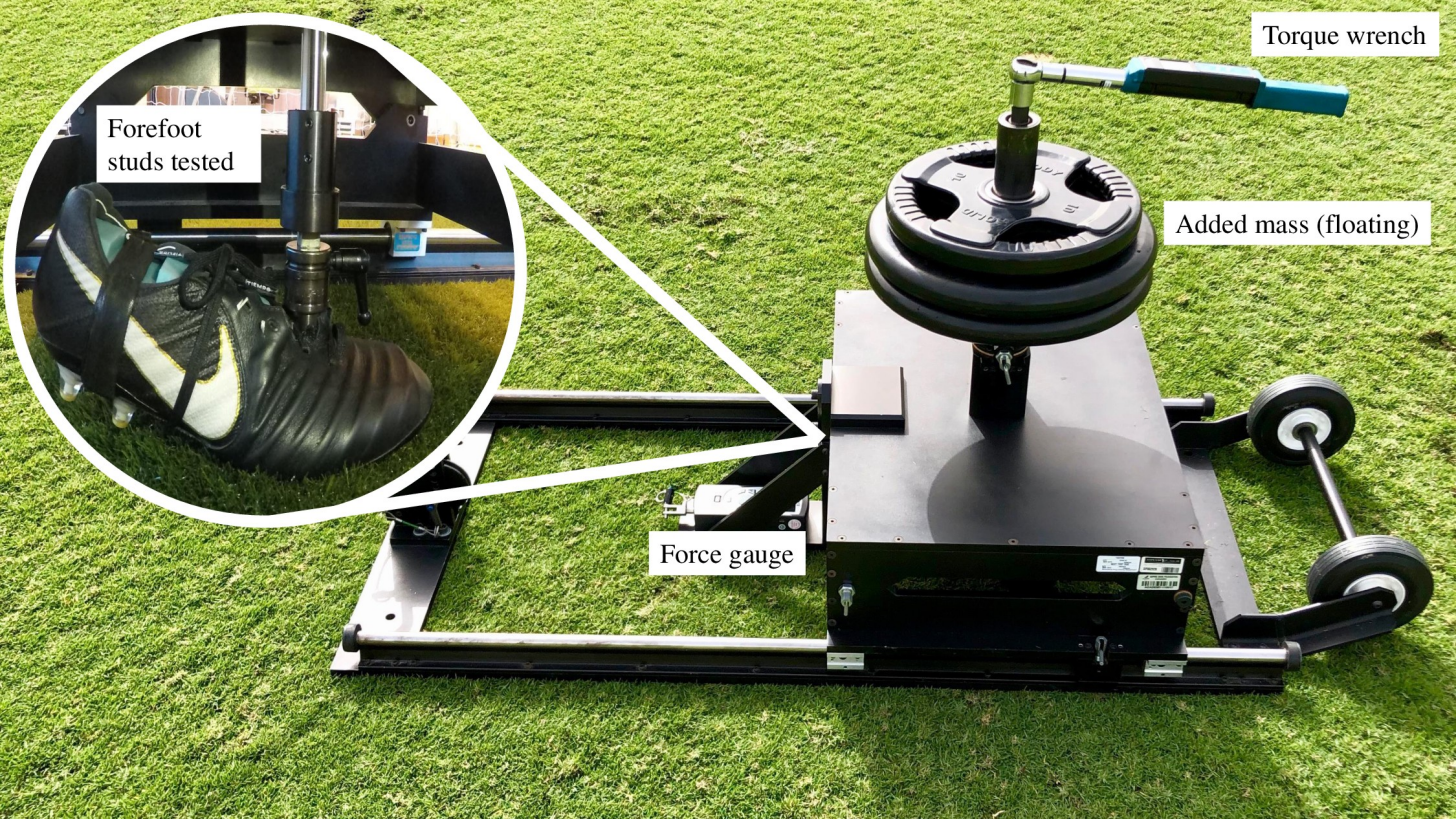
Portable shoe-surface traction testing machine. (S2T2, Exeter research, USA).

Traction between the shoe and surface was measured using a commercially available portable traction testing device (S2T2, Exeter Research USA). The device consists of a prosthetic foot-form (size 10.5 US), on which shoes are fitted and positioned at 20° of plantar flexion to ensure only the forefoot studs are in contact with the surface [25,26]. The foot can be rotated to measure peak rotational traction or locked into a linear position along the long axis of the shoe and then dragged forward across the surface to measure translational traction [10,26]. The floating foot-mass ensures the vertical load (added barbell weight plates) is applied through the shoe to the playing surface and not the supporting frame. Wheels allow for movement across multiple testing locations on the playing surface (Fig 2). Measurements were taken manually by a single operator (AT) for all pilot validation tests and within study tests. Each shoe model was tested at twelve separate locations on the playing surface during the five individual time points (November 2017, January, March, April and May 2018) for rotational traction and six separate playing surface locations for translational traction.

For rotational traction a vertical load of 580N (59.1kg) was applied and the test foot rotated through 90° at a speed of approximately 90°/s. Two operators who had a combined mass of 163kg stood on each end of the frame to stabilise during tests. Peak rotational traction was recorded in newton meters (Nm) for both internal rotation and external rotation directions by a digital torque wrench sampling at 500Hz (ETW-PR-100, Checkline, NY, USA) with an accuracy of ± 1% of indicated measurement in a range of 10-100Nm. Rotational traction and vertical load displayed a linear relationship during our pilot work with the S2T2 tester on this natural grass playing surface as previously reported [26]. Thus, 580N was deemed to cause an acceptable amount of damage for grounds-staff to manage on a high use football surface and is a vertical load used for previous studies in American football [10, 26].

For translational traction a vertical load of 300N (30.6kg) was applied to the test foot while a digital force gauge (Chatillon DFE2-500, Ametek, USA) sampling at 7000Hz with an accuracy of ±0.25% of indicated measurement, measured peak horizontal force (Newtons) resisting linear motion between the shoe and surface. The translational traction coefficient was calculated as a ratio of peak horizontal force divided by vertical force. This gives an indication of the horizontal force required to overcome the resistance between the shoe and surface as the shoe is dragged across the surface in a linear movement. During pilot work several speeds and vertical loads were used for translation traction testing with ground-staff present to assess damage to the playing surface. 300N of vertical load and approximately 200mm/s allowed surface damage acceptable to ground staff.

### Reliability and validity of shoe-surface traction tester

A test-retest protocol comprising 528 measurements of a single football pitch was conducted for internal rotation, external rotation, and translational traction, prior to commencing data collection for the current study. Intra-class correlation coefficients with 95% confidence intervals, standard error of measurement (SEM), and minimal detectable change (MDC) are presented in Table 1.

**Table 1:** Intra-class correlation coefficients (ICC) with 95% confidence intervals, standard error of measurement (SEM), and minimal detectable change (MDC) for internal rotation, external rotational traction (in Newton meters), and translational traction (in Newtons). ICC values were classified as follows; ≥0.9 as excellent, ≥0.8 as good, ≥0.7 as acceptable, ≥0.6 as questionable, ≥0.5 as poor and <0.5 as unacceptable [27].

	ICC(95%CI)	SEM	MDC (%)
**Internal Rot**	0.94 (0.91–0.96) Excellent	1.8	5(12%)
**External Rot**	0.94 (0.91–0.96) Excellent	1.8	5 (12%)
**Translation**	0.76 (0.67–0.86) Acceptable	35	98 (17%)

### Statistical analysis

The dependent variable was rotational traction. A 2-way analysis of variance, was conducted using two factors: month/surface (5 levels) and shoe model (6 levels). Bonferoni post hoc tests were performed when indicated. This analysis was repeated to further examine the effect of month / surface (5 levels) and outsole pattern (3 levels). We also undertook a series of ANCOVAs. This was to compare main and interaction effects after controlling each of the following covariates which were dichotimsed using medians of temperature, humidity and ground hardness. All statistical tests were undertaken using SPSS (Version 25, IBM, Chicago, Illinois) with significance was set at P < .05 in all analyses.

## Results

### Rotational traction

Table 2 summarizes the mean rotational traction in newton meters (Nm) for individual shoe models at each testing time point with grass type in bold. Peak rotational traction was significantly different across shoe models (F = 218, df = 5, p <0.0001). Consistently lower rotational traction was recorded with the Tiempo AG shoe across all months. Post hoc testing found significant differences between the Tiempo SG and all other models, with the largest difference occurring between the Tiempo SG (metal screw-in studs) and Tiempo AG (small round moulded studs) shoes (Mean difference 17.5 Nm, t = 13.3, p<0.0001). Consistently higher rotational traction was recorded for the Tiempo SG shoe across all months.

**Table 2:** Mean rotational traction in Newton meters (Nm) for individual shoe models at each testing time point with grass type in bold. WS = warm season grass. CS = Cool season grass. WS/CS = warm season grass over-sown with cool season grass. Outsole type groups AG = artificial grass, FG = firm ground and SG = Soft ground. Conditional formatting shows the minimum (green) and maximum (red) rotational traction shoe-surface combinations with the highest (dark red) being Tiempo SG shoe tested in May on warm season grass. Winter is December-February in Qatar hence the cool season grass and Tiempo AG shoe combination in January showed the lowest mean rotational traction (dark green).

		Month/Grass type						
Shoe	Outsole	Nov WS	Jan CS	March CS/WS	April WS	May WS	Mean	SD
Tiempo	AG	36.1	28.1^c^	34.8	37.2	42.3	35.7^a^	5.1
Hypervenom	FG	45.6	32.3	40.8	39.3	46.3	40.9^a^	5.7
Tiempo	FG	50.4	34	44	43.6	45.7	43.8^a^	6.0
Magista	FG	49.8	38.5	43	47.2	49.7	45.7^a^	4.9
Mercurial	FG	49.6	40.4	40.8	48.3	54	46.2^a^	5.8
Tiempo	SG	56.6	44.6	53.5	52.2	59.1^c^	52.2	6.2
	Mean	48.0	36.3^b^	42.8 ^b^	44.6	49.5		
	SD	6.8	6.0	6.1	5.7	6.1		

^a^ Significant difference from Tiempo SG shoe.

^b^ Significant difference from November warm season grass.

^c^ Largest mean difference

Significance P < .05

Peak rotational traction was also significantly affected by grass type (F = 202.8, df = 4, p < 0.0001). Colder season grass (January) was associated with the lowest rotational traction. Conversely, the highest values were reported when testing on warm season grass (during either November or May testing). The largest mean differences occurred when comparing May warm season grass (WS) vs. January cool season grass (CS) (Mean difference 13.2 Nm, t = 10.9, p<0.001). (Table 2 and S1 Fig). Large differences in rotational data were also reported when comparing January CS with November WS (Mean difference 11.7 Nm, t = 9.7, p<0.001). We also found a significant interaction between the two factors (month*shoe type) (F = 5.4, DF = 20, p <0.0001).

**Fig 3:**
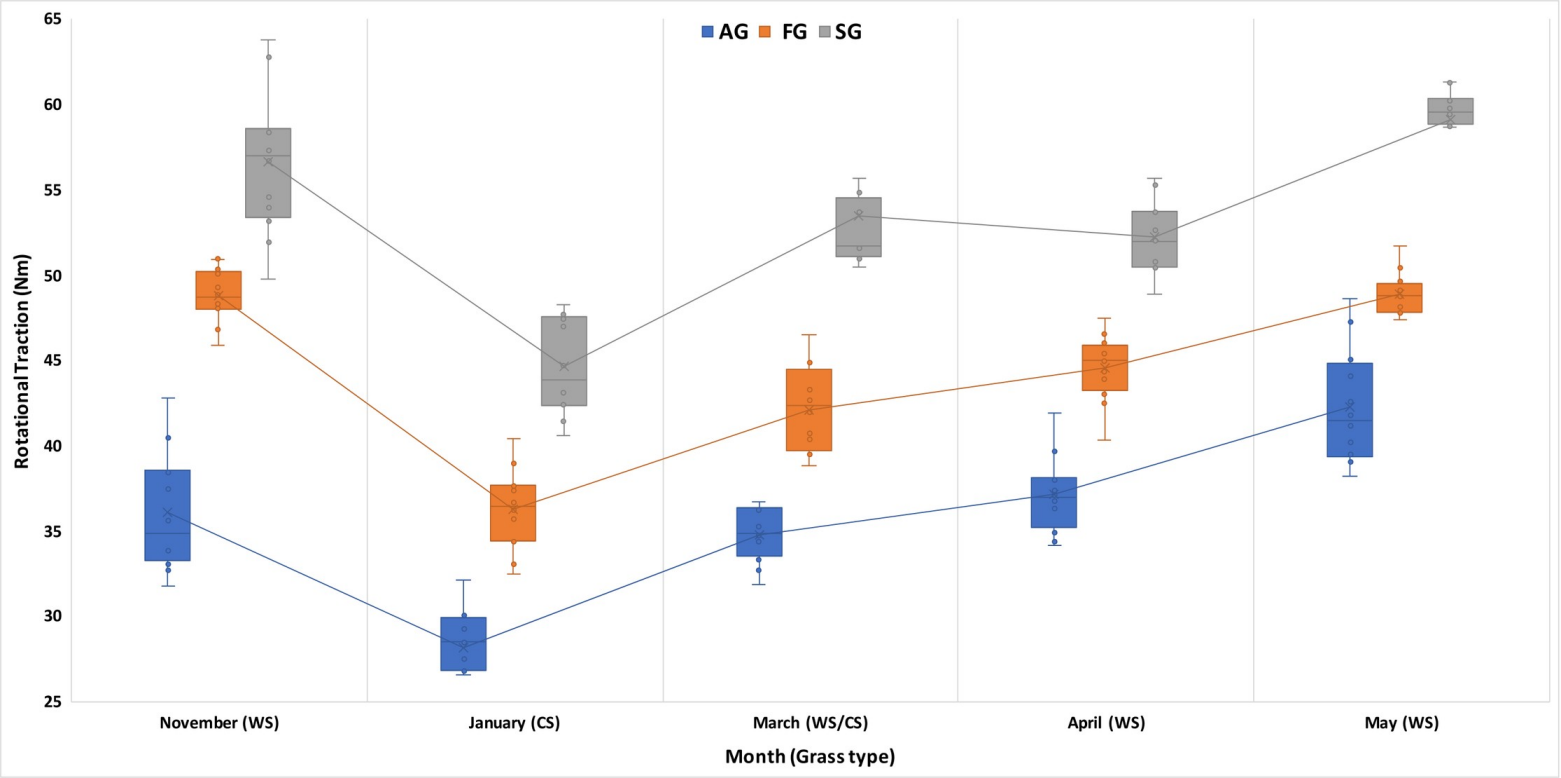
Rotational traction (Nm) for outsole type at each testing time point. WS = warm season grass. CS = Cool season grass. WS/CS = warm season grass over-sown with cool season grass. The box represents 50% of the dataset, ends of the box show the 1st and 3rd quartiles, whiskers extend to the furthest data point within 1.5*IQR from the 1st and 3rd quartiles. ‘X’ within box = mean. Horizontal line within box = median. Whiskers of artificial ground (AG) blue outsole never cross that of the Soft ground (SG) grey outsole type for the entire season. Note the relative drop in rotational traction for all outsole groups in January with the cool season grass playing surface.

Findings were similar when analyses were repeated using outsole classification (rather than shoe model). Again, we found significant main effects for outsole (F = 316.2, df = 2, p < .0001), grass type (F = 87.1, df = 4, p < .0001) and significant interaction effects (outsole*grass type) (F = 2.7, df = 8, p < .007). The largest mean difference for rotational traction was reported in November (warm season grass) between the SG and AG outsoles– 20.6Nm (*17*.*3–23*.*8* 95%CI) with very large effect size (ES = 5.4). See Table 3 and Fig 3.

**Table 3:** Mean difference (95% CI) for rotational traction (Nm) and *effect size (95%CI)* for different shoe outsole types at each testing time point and grass type (bold). WS = warm season grass. CS = Cool season grass. WS/CS = warm season grass over-sown with cool season grass. Note the consistently large mean differences with very large effect size between the soft ground (SG) and artificial ground (AG) outsoles across the entire season.

	Month/Grass type					
	November WS	January CS	March WS/CS	April WS	May WS	
FG vs AG	12.8 (10.5–15.0)	8.2 (5.3–10.9)	7.3 (4.9–9.1)	7.5 (4.7–10.2)	6.7 (4.3–9.1)	Mean Difference (95% CI)
	*3*.*7 (2*.*8–4*.*6)*	*1*.*9 (1*.*1–2*.*5)*	*1*.*9 (1*.*2–2*.*6)*	*1*.*8 (1*.*1–2*.*5)*	*1*.*8 (1*.*1–2*.*5)*	*Effect Size (95% CI)*
SG vs FG	7.8 (5.5–10.1)	8.4 (5.5–11.2)	11.3 (8.6–14.1)	7.6 (4.9–10.3)	10.2 (7.8–12.5)	
	*2*.*2 (1*.*4–2*.*8)*	*1*.*9 (1*.*2–2*.*6)*	*2*.*7 (1*.*9–3*.*5)*	*1*.*8 (1*.*1–2*.*5)*	*2*.*8 (1*.*9–3*.*6)*	
SG vs AG	20.6 (17.3–23.8)	16.5 (14.2–18.8)	18.6 (16.1–21.2)	15.1 (13.2–16.9)	16.8 (14.2–19.5)	
	*5*.*4 (3*.*5–6*.*8)*	*6*.*2 (4*.*1–7*.*8)*	*6*.*1 (4*.*1–7*.*8)*	*7*.*0 (4*.*7–8*.*9)*	*5*.*4 (3*.*6–7*.*0)*	

Table 4 shows the average values for climate and surface tests. Exploratory analyses (ANCOVA) found trends that lower humidity (B = -2.4, t = -1.9, p = .06) and greater ground hardness (B = 2.5, t = 1.9, p = .052) were associated with higher rotational traction. Higher temperatures were also associated with higher levels of rotational traction (B = 3.18, t = 2.5, p = .012). There was still a significant effect of shoe type and shoe*grass interaction, on levels of rotational traction after controlling for each of these covariates. Temperature was not included as a covariate when assessing the main effects of grass type due to the high level of correlation between these two variables. (r = .88, p < .0001).

**Table 4:** Average values for climate and surface tests conducted at five pitch locations and repeated five times at each location (move to unaffected grass for each test).

	Month/Grass type						
	November WS	January CS	March WS/CS	April WS	May WS	Mean	SD
Temperature (°C)	29	22	27	32	35	29	5
Humidity (%)	59	35	60	34	28	43	15
Soil Moisture (%)	25	24	21	19	21	22	2
Surface Hardness (g)	72	64	78	71	80	73	6

### Translation traction

No main effect by shoe model was found for translational traction (F = 2.392, p = 0.07). However, there was a main effect for grass type (F = 3.861, p = 0.01) with the largest difference being warm season grass and Tiempo SG combination (translational traction coefficient mean μ = 2.5±0.2) vs the cool season grass and Tiempo AG combination (translational traction coefficient mean μ = 1.9 ±0.1). The translational traction coefficient was calculated as a ratio of peak horizontal force divided by vertical force.

## Discussion

Large variations in the magnitude of shoe-surface traction are evident throughout one season of elite football played in a warm/hot climate. Shoe type, outsole group, and grass species significantly affected rotational traction which has been linked to increased lower extremity injury [8–11,18]. Implications for footwear selection will interest players, medical and sports science staff working in football played in warm climate zones, particularly when it is vital to minimise rotational traction for given playing surfaces and climate conditions (e.g. return to on-field rehabilitation in football shoes after ACL injury).

The major strengths of this study include data collection of different shoe outsole designs, grass species, surface mechanical properties, and climate data at multiple time points on a playing surface maintained for elite football (not a turf farm or laboratory setting).

### Why is shoe-surface traction important?

While performance may be augmented with higher available traction at the shoe-surface interface, some concerning alterations to player movement can occur. Lower knee flexion angle, higher external knee valgus moments, increased knee joint loading, and increased distance from the plant foot to the centre of mass during cutting manoeuvres are some of the changes under higher traction conditions at the shoe-surface interface [7, 12, 22]. These movement strategies, along with increased loading, have been implicated in anterior cruciate ligament (ACL) injury and other lower extremity injuries. This is corroborated with evidence from prospective studies showing a significant increase in lower limb injury risk associated with high levels of rotational traction [8–11]. Importantly, higher rotational traction, as opposed to translational traction, has been found to be a significant predictor of peak ACL force during a maximal change of direction task [28].

### What can players do to modulate rotational traction?

Parameters that are somewhat set once the player arrives to train or play a match include the climate, surface hardness, surface traction, and grass type etc. Pitch preparation and climate are also out of the athlete’s control. Shoe outsole selection is one of the few immediately modifiable factors that can allow a player to modulate the traction experienced at the shoe-surface interface [5]. Objective data on the surface should be made available to the athlete and medical or sport science teams so that footwear selection can be made with these parameters in mind. Significant differences for rotational traction were found at the shoe-surface interface for different grass species (Table 2), shoe types (Table 2, S1 Fig), and shoe outsoles (Fig 3).

Overall, choosing a shoe with lower rotational traction that results in no consequent detriment to performance (high translational traction) is recommended, assuming the injury risk from other sports extends to soccer [7–10, 29]. Table 2, S1 Fig, and Fig 3 can be used to help inform footwear selection for players in warm climate zones.

### Stud/cleat shape and surface conditions

Players deem optimal performance and/or risk of lower extremity injury to be intrinsically related to certain playing surface characteristics. Ninety-one percent of players from a worldwide cohort of elite footballers (n = 1129) think the type or condition of a playing surface increases the likelihood of injury with excessive hardness and traction ranked high on the list of concerns [30].

Optimum penetration of the stud/cleat into the surface is paramount in achieving the maximum traction [19] (which is beneficial to performance) as all studs ‘sink’ into the surface to the outsole plate. Surface hardness therefore affects traction and comfort for the player depending on the type of shoe outsoles used. Ground staff kept the soil moisture and surface hardness within a small range of variation across each individual testing point in this study (Table 4). The SG outsole had the highest values for both rotational and translational traction over the season. Conical, tapered metal screw-in studs 11mm in length at the forefoot of the SG outsole allow for full penetration in the playing surface and significantly increase traction. This is evident in May with high surface hardness, high temperature, warm season grass, and the SG outsole combined to give the highest mean peak rotational traction for the season (59Nm). Some of the FG shoes with bladed cleats had a larger cross-sectional area than the tapered conical studs of the SG shoe (Fig 1) and may not have penetrated completely to the outsole plate of the shoe and therefore demonstrated lower rotational traction values. However, the penetration depth of studs was not measured in this study.

### Effect of grass type on rotational traction

Warm season (Paspalum) grass showed higher rotational and translational traction particularly when coupled with the SG outsole. Cool season (Rye) grass showed lower rotational traction across all shoes highlighted by the relative “dip” in rotational traction values for all outsole types in January (Fig 3) compared to other months where there is either warm season grass or warm season grass over-sown with cool season grass. Our findings add a mechanically plausible explanation to those of Orchard *et al*. (2005) [24] in which male Australian rules football players suffered less ACL injuries on cool season Rye grass than warm season (Bermuda) grass.

### Considerations for return to field specific rehabilitation following injury

Rotational traction for the AG outsole was consistently lower regardless of grass type, climate, and mechanical properties (e.g. hardness) of the pitch (Fig 3). We suggest this should be the outsole of choice for those players returning to on-field sports specific rehabilitation following ACL, syndesmosis or other lower extremity injuries where it is vital to minimise rotational traction forces. Conversely, SG metal screw-in studs consistently showed high rotational traction and should ideally be avoided during early stage field specific rehabilitation.

Lambson *et al*. (1996) [8] investigated the effect of cleat design on ACL injury risk in American football. Large cleats located along the edge of the forefoot in American football shoes were shown to have higher rotational traction (average 31Nm). Subsequently, 3.4 times more ACL injuries occurred with this cleat design than other stud or cleat designs that had lower rotational traction values (average 24Nm). Comparisons are difficult as we tested with higher vertical load, on surfaces with different characteristics, and used a commercially available traction-testing machine. Wannop *et al* (2013) [10] used the same vertical load as our study (580N or 60kg) on a more sophisticated traction testing machine to investigate the effect footwear traction has on lower extremity injury in American football. Non-contact lower extremity injuries peaked at 19.2/1000 game exposures in the high rotational traction group (39–54.9Nm) of male American football players compared to 4.2 injuries per 1000 games exposures in the low rotational traction group (15–30.9Nm). Prospective studies are of course required to see if these findings extend to soccer.

### Translational traction testing on natural grass playing surfaces

Remarkably, only grass type affected translational traction. There was no main effect relating to translational traction seen for shoe type or outsole type. This was a surprising finding as there was considerable damage to the playing surface with each test. Previous research on the coefficient of translational traction tested on artificial playing surfaces suggests vertical loads of over 888N (approx. 90kg) are required to see meaningful differences between shoe outsole designs when a horizontal force is applied [31]. Our findings suggest the vertical load of 300N used here was not sensitive enough to see differences between the shoes tested on two species of natural grass. It was not feasible to test at higher loads due to the amount of damage incurred to the playing surface with each test. The playing surface examined was the Qatar national team’s main training pitch which saw high traffic over the duration of the study. Speed of the horizontal translation (which was manually driven) may also have influenced the results [26]. It is suggested that improved and more sensitive methods for testing translational traction need to be developed if it is to be implemented into regular monitoring at elite football clubs and federations.

### Does mechanical traction testing equal traction utilised by a player?

In January cool season grass average peak rotational traction for the AG outsole slipped down to 28Nm compared to 36Nm and 45Nm for the FG and SG groups respectively (Table 3). Further biomechanical and perception testing of players performing football specific tasks are required to ascertain if performance decreases with this lower magnitude of rotational traction [5].

Our results suggest mechanical testing for traction at the shoe-surface interface is more sensitive to changes in the rotational component of traction compared to the translational component for the methods used here.

### Limitations

Although portable testing devices facilitate tracking of surface properties over time and between different surfaces or different football shoes, they do not provide an accurate representation of forces experienced by players when they are actually playing sport. It is suggested that a functional traction course and traction perception rating be used alongside mechanical testing to allow players’ intuition and perception of optimal traction to aid footwear selection [5].

All shoes tested here are from one manufacturer. Future research should test across all football shoe manufacturers.

### Impact of our findings

After ground staff have prepared a playing surface and the prevailing climatic conditions are known close to kick-off, thereafter players can only control the type of shoe outsole (e.g. soft ground outsole, firm ground outsole etc) by choosing the shoe that best suits these primary factors to modulate the amount of traction experienced by the player. The current data shows that the variability within a single season is large enough to warrant tailoring across different months.

### Further research

It is likely that the optimal level of traction may change based on sport or even playing position. It is also pragmatic to suggest even lower levels of rotational traction when players are returning to field specific rehabilitation or training following a significant injury (eg ACL). Future research should examine several playing surfaces, soil types, and grass species to get a more complete understanding of shoe-surface traction.

## Conclusions

The rotational (but not translational) traction varied substantially across different months of the year, different grass species, and with different shoe outsole types. Warm season grass tested with the soft ground shoe (screw-in metal studs) showed the highest magnitude of rotational traction while cool season grass tested with an artificial ground shoe (small round moulded studs) showed the lowest. These objective data should allow for more informed footwear choices for football played in warm/hot climates. Further research is required to confirm if these findings extend across other football shoe brands.

### Practical implications

Objective data presented here can help tailor footwear selection (from one manufacturer) across a season of elite football in warm/hot climate zones. The authors suggest a universally accepted (commercially available) portable shoe-surface traction device should be agreed upon to allow new footwear outsole designs to be tested on various playing surfaces and climate zones.

## Supporting information

S1 Fig Rotational traction for each shoe at each testing time point and grass type. WS = warm season grass. CS = Cool season grass. WS/CS = warm season grass over-sown with cool season grass. The box represents 50% of the dataset, ends of the box show the 1st and 3rd quartiles, whiskers extend to the furthest data point within 1.5*IQR from the 1st and 3rd quartiles. ‘X’within box = mean. Horizontal line within box = median.
(TIF)

S1 File Dataset of shoe-surface traction tests. (XLSX)
