# Correction: Six different football shoes, one playing surface and the weather; Assessing variation in shoe-surface traction over one season of elite football

**DOI:** 10.1371/journal.pone.0218865

**Published:** 2019-06-19

**Authors:** Athol Thomson, Rodney Whiteley, Mathew Wilson, Chris Bleakley

The following information is missing from the Funding statement: The publication of this article was funded by the Qatar National Library.
