# Supplementary figures and images for: Six different football shoes, one playing surface and the weather; Assessing variation in shoe-surface traction over one season of elite football
Source: PLoS One. 2019 Apr 30;14(4):e0216364. doi: 10.1371/journal.pone.0216364 (PMC6490939; doi:10.1371/journal.pone.0216364)

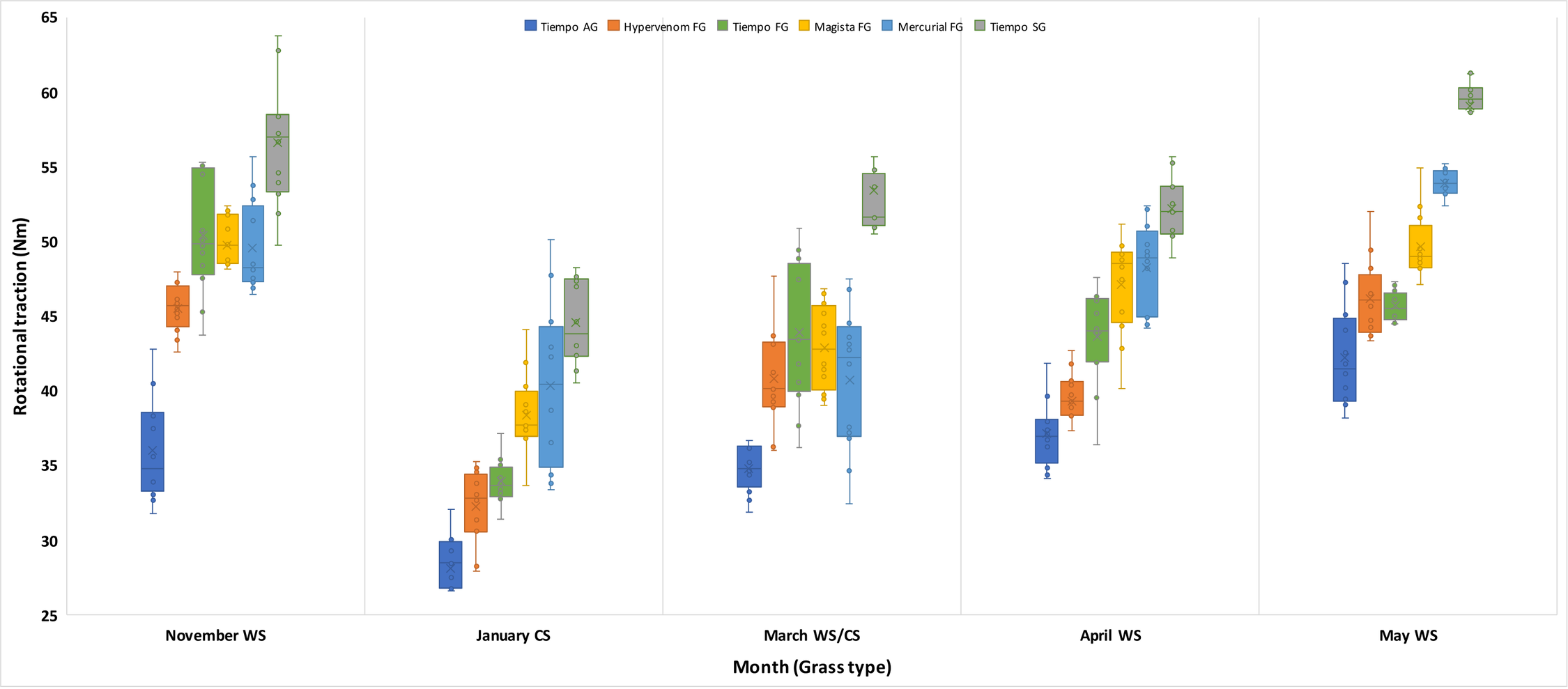

Supplement: S1 Fig — WS = warm season grass. CS = Cool season grass. WS/CS = warm season grass over-sown with cool season grass. The box represents 50% of the dataset, ends of the box show the 1st and 3rd quartiles, whiskers extend to the furthest data point within 1.5*IQR from the 1st and 3rd quartiles. ‘X’within box = mean. Horizontal line within box = median. (TIF) [file pone.0216364.s001.tif]
